# Gerontome: a web-based database server for aging-related genes and analysis pipelines

**DOI:** 10.1186/1471-2164-11-S4-S20

**Published:** 2010-12-02

**Authors:** Jekeun Kwon, Byungwook Lee, Haeyoung Chung

**Affiliations:** 1 Korean BioInformation Center (KOBIC), KRIBB, Daejeon 305-806, Korea; 2 Interdisciplinary Research Program of Bioinformatics, Busan National University, Busan 609-735, Korea; 3 Aging Tissue Bank, College of Pharmacy, Busan National University, Busan 609-735, Korea

## Abstract

**Background:**

Aging is a complex and challenging phenomenon that requires interdisciplinary efforts to unravel its mystery. Insight into genes relevant to the aging process would offer the chance to delay and avoid some of deteriorative aspects of aging through the use of preventive methods. To assist basic research on aging, a comprehensive database and analysis platform for aging-related genes is required.

**Results:**

We developed a web-based database server, called Gerontome that contains aging-related gene information and user-friendly analysis pipelines. To construct the Gerontome database, we integrated aging-related genes and their annotation data. The aging-related genes were categorized by a set of structural terms from Gene Ontology (GO). Analysis pipelines for promoter analysis and protein-ligand docking were developed. The promoter analysis pipeline allows users to investigate the age-dependent regulation of gene expression. The protein-ligand docking pipeline provides information on the position and orientation of a ligand in an age-related protein surface.

**Conclusion:**

Gerontome can be accessed through web interfaces for querying and browsing. The server provides comprehensive age-related gene information and analysis pipelines. Gerontome is available free at http://gerontome.kobic.re.kr.

## Background

Aging is universal phenomenon among all organisms. Because the processes underlying aging are controversial and it is a poorly understood biological problem, aging-related genes have attracted a fair amount of attention from both the academic community, the medicinal community and the public in general [1]. Aging is a risk factor for many diseases [2]. Many studies have been performed in several model organisms, including humans, to obtain new insights into the process of aging and to identify aging-related genes by comparing young and old tissues or by comparing samples across a lifespan [3]. Information on genetic links to cellular aging suggests new treatments for a variety of age-related diseases and cancers [4].

A collection of age-related information in multiple organisms is important to understand complicated age phenomenon and to identify new age-related data. Several age-related databases have been constructed based on gene, protein, or microarray experiments. The Human Aging Genomic Resource (HAGR) [5] provides manually-curated aging genes in human and model animals. Gene Aging Nexus (GAN) [6] contains aging-related gene expression patterns in multiple organisms under different conditions. The aging genes and interventions database (AGEID) [7] provides experimental results related to aging and information on genes that influence the incidence of age-associated disorders such as Alzheimer's disease. However, the efficient exploitation of this large data set is hampered by the lack of an integrated database and data analysis platform.

Here we have constructed a database server, called Gerontome, to provide comprehensive information on aging-related genes and analysis interfaces. We integrated aging-related resources and developed automated analysis pipelines to provide transcription factor binding sites of regulatory regions and docking information between proteins and ligands in aging-related genes. We categorized aging-related genes by a set of structural terms from Gene Ontology (GO). Our aim in building Gerontome is to provide researchers with a comprehensive online resource and a user-friendly analysis interface to study the genetic basis of aging.

## Methods

### Data sources

Aging-related gene information was obtained from HAGR (http://genomics.senescence.info/), AGEID (http://uwaging.org/genesdb/index.php), the meta-analysis of age-related gene expression Profiles [3], and aging-related yeast2hybrid experiment [8]. From the downloaded data, we created a non-redundant gene set by removing the redundancy in the three databases. As of April 1, 2010, the Gerontome database had 848 non-redundant aging-related genes.

**Figure 1:**
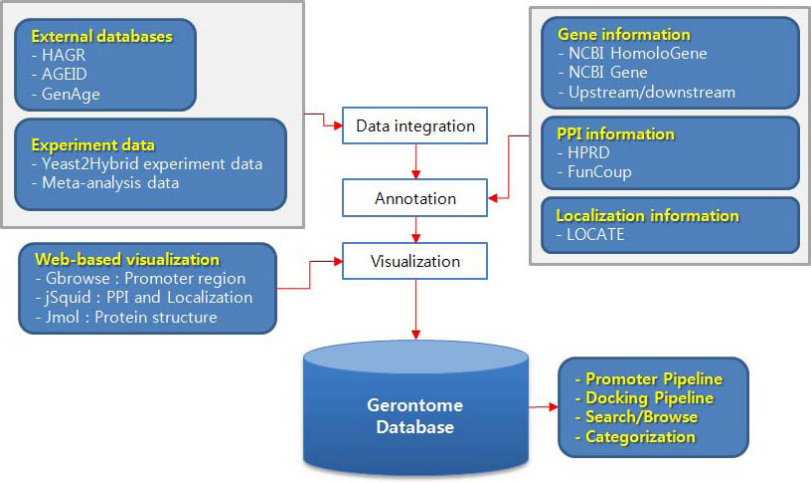
Flowchart of Gerontome database construction.

Gerontome uses data from a number of other databases. Human homologs were downloaded from NCBI’s Homologene [9]. Promoter sequences of human genes were obtained from the UCSC genome browser (hg18) [10]. Transcriptional profiles and protein-protein interactions were taken from the Transcription Factor Binding Site (TFBS) conserved track in the UCSC genome browser [10] and HPRD [11] databases, respectively. We used the LOCATE database [12] to identify localization information and the Funcoup database [13] to obtain confidence scores of protein-protein interactions. These data were mapped into aging-related genes and integrated into the Gerontome database (Fig. 1).

We used Gene Ontology (GO) annotation, which describes how gene products behave in a cellular context [14]. GO is composed of three subdivisions covering basic areas of biological research: molecular function, biological process, and cellular function. To identify GO categories that tend to be associated with aging genes, we used files downloaded from Entrez Gene database [15]. Through the categorization, we were able to assign 848 genes to GO accession numbers.

### Analysis pipelines

Gerontome provides information regarding the molecular features of aging-related genes such as transcription factor binding sites and protein-ligand docking. To provide this information, we developed two analysis pipelines: promoter analysis and protein-ligand docking.

**Figure 2:**
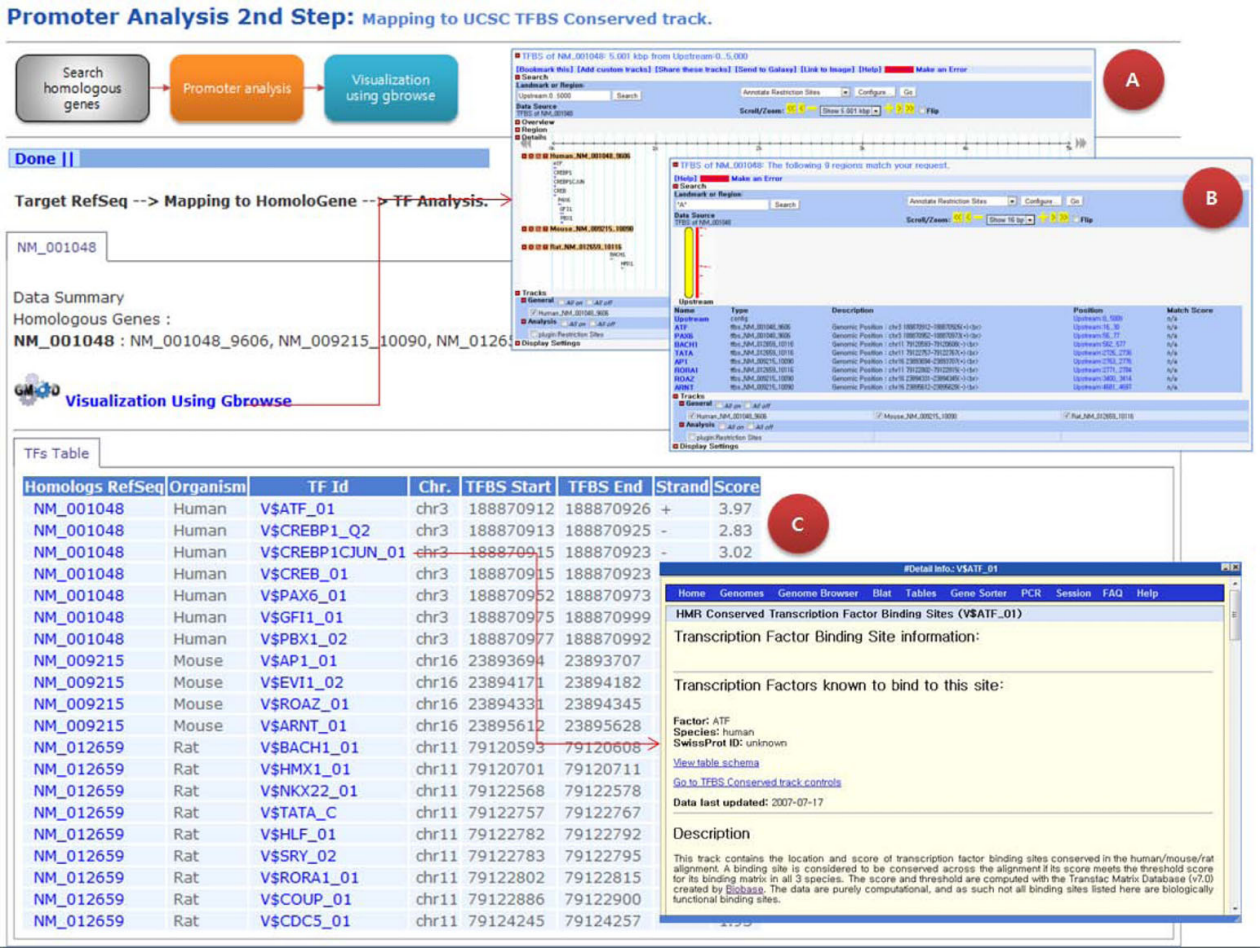
**Comparative transcriptional regulatory network by promoter pipeline.** The pipeline provides step-by-step processes with the user’s own data. In the pipeline, a user can obtain information such as A) a visualization of promoter regions, B) a list of TFBS, and C) detailed information about TFBS.

The promoter analysis pipeline allows users to investigate the age-dependent regulation of gene expression through the identification of transcription factors and their binding sequences (Fig. 2). Identification of transcriptional regulation of age-related genes is generally the most important step in aging research. In the pipeline, homologous genes to the query identifiers were first extracted from NCBI's Homologene. Second, upstream sequences of the extracted homologous genes were obtained. The default length of upstream sequences was set at 1000 bases. Third, the server scanned transcription factor binding sites in the upstream sequences using the TFBS conserved information from the UCSC genome browser. Lastly the server provided comparative visualization of homologous genes, TFBSs information, and known genes. We used Gbrowse [16] to visualize the results. From the pipeline, users can find a correlation between age-related genes and transcription factor binding sites [17-19].

**Figure 3:**
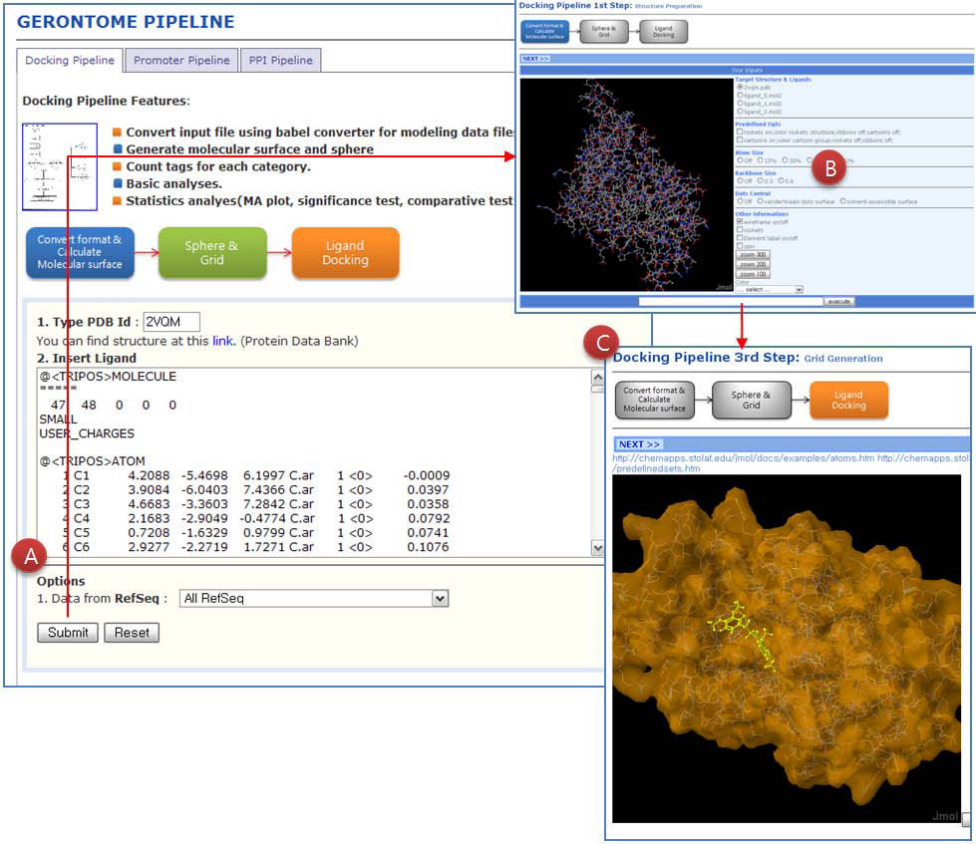
**Docking analysis between proteins and ligands.** A) PDB number and ligand text format were used to run docking simulations and visualization. B) Docking between protein structures and ligands were visualized by the Jmol program. C) Users can find additional information about the affinity and activity of small molecules.

The identification of protein structure is a key step to understanding the biological function and biomolecular interactions of proteins. Docking between proteins and ligands is important in the development of anti-aging drugs. Docking is the identification of the low-energy binding modes of a small molecule or ligand within the active site of a macromolecule or receptor whose structure is known. In the protein-ligand docking pipeline, the positions and orientations of ligands in protein surfaces were predicted using a geometric matching algorithm in the Dock Program [20] (Fig. 3). Users can dock their ligands to surfaces of protein structures. To view ligand positions on protein structures, we used a JMol program [21]. The protein-ligand docking pipeline enables users to simulate interaction affinity without ligand information or a specific protein structure.

### Web-based server

We developed a web-based server to provide a back-end pipeline for aging analysis and to allow users to compare their genes and proteins with the Gerontome database. The Gerontome database server is composed of a wiki-based web interface and a MySQL 5.0 database management system. The web interface is implemented in static HTML pages, PHP, and JavaScript under an Apache 2.2 web server. MySQL is used to store the age-related gene information and their annotations and analysis data.

## Results and discussion

**Figure 4:**
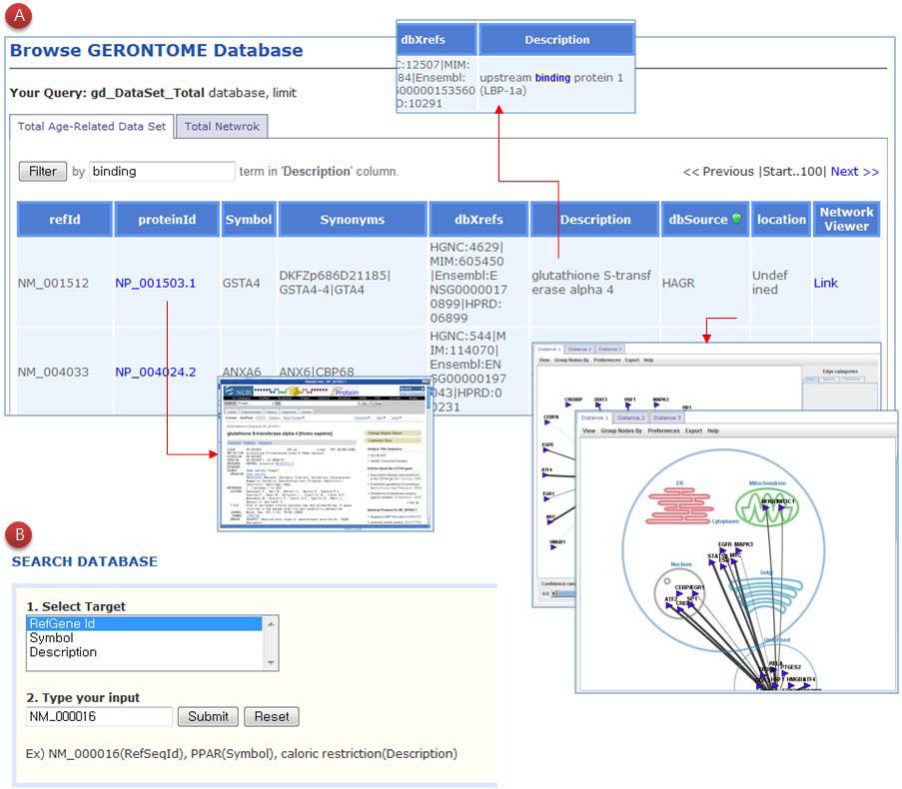
**User interface for search and browse.** A) Users can choose a database source from Total, AGEID, HAGR, and Y2H. Users can filter by term, network visualization, and other options. B) Gerontome also provides various keyword inputs such as RefSeq number, gene symbol, and description.

Gerontome can be accessed through a web interface for querying and browsing (Fig. 4). The querying interface allows the user to search against age-related genes and their annotation data. Age-related genes can be searched by RefSeq [22] number, gene symbol, and description. The search results contain basic information, observations, phenotypes, and gene expression data for aging. In the browsing interface, the user can select an aging data source and then see all of the aging-related data. In the browsing results, the user can filter by description term.

We also categorized the aging-related genes by a set of structural terms from Gene Ontology (GO). The user can see the categorized genes by clicking on ‘categorization of age-related data’ and download GO mapping results for biological process, cellular component and molecular function at our site. From the GO mapping results, we found that age-related genes are highly related to 'regulation of transcription', 'anti-apoptosis', 'apoptosis', and 'response to DNA damage stimulus' in the biological process category (Table 1). In addition we used the Gene Ontology Enrichment Analysis Software Toolkit (GOEAST) [23] for GO enrichment analysis. Users can browse the GO enrichment analysis results.

**Table 1:** Classification of age-related genes according to Gene Ontology(GO) terms.

**Order**	**Cellular component**	**No. genes**	**Molecular function**	**No. genes**	**Biological process**	**No. genes**

1	Nucleus	408	protein binding	435	regulation of transcription, DNA-dependent	117
2	cytoplasm	391	nucleotide binding	160	signal transduction	98
3	membrane	163	metal ion binding	137	transcription	82
4	plasma membrane	155	zinc ion binding	131	anti-apoptosis	48
5	extracellular region	150	ATP binding	123	cell cycle	47
6	cytosol	137	transferase activity	95	interspecies interaction between organisms	44
7	mitochondrion	107	transcription factor activity	85	apoptosis	44
8	integral to membrane	107	DNA binding	78	response to DNA damage stimulus	43
9	intracellular	106	calcium ion binding	64	multicellular organismal development	42
10	nucleolus	86	hydrolase activity	56	cell adhesion	38

Gerontome provides several viewers for the TFBSs position, protein structure, and protein interaction of each entry by using Gbrowse, jSquid [24], and JMol programs. In the Gbrowse interface, users can compare biological features between homologous genes and proteins which represent relatively closed protein groups. jSquid displays the protein-protein interaction network among age-related proteins. In the jSquid search results, users can modify subgroups of network elements based on the annotation information on protein localization and the confidence score of protein-protein interaction. After docking between aging-related protein and ligands, users can see the position and orientation of a ligand in an age-related protein surface by using JMol, which is a Java viewer for chemical structures in 3D with features of bio-molecules and materials.

In addition, we developed a wiki site for sharing information about Gerontome. The wiki aims to promote sharing information and knowledge among researchers. The wiki also includes detailed information on the analysis pipelines, the parameters of programs, and a data summary of our database. The Gerontome wiki is available at http://www.gerontome.info/wiki/index.php.

## Conclusion

We developed a database and tools that will be useful to researchers working on the science of aging. Our aim is for Gerontome to become a major resource for understanding the systematic mechanisms of human aging. To facilitate the integrative analysis of aging genes, we constructed a comprehensive aging gene database and developed a web-based analysis platform, which is freely accessible to the research community to query, analyze, and visualize age-related genes. The database also has links to genomic information from different species to facilitate the discovery of candidate genes that are involved in aging through a genome-wide comparative analysis. The analysis pipelines in Gerontome are useful to predict regulatory networks of homologous genes, docking simulations between protein structures and ligands, and protein interaction networks.

In the future, we will upgrade, update and expand the resources in Gerontome as well as develop new tools that can benefit the gerontology community. The aging gene information in the Gerontome will be useful when trying to identify new treatments and drugs for a variety of age-related diseases. We would like Gerontome to become a general platform for bio-gerontologists and bioinformaticians.

## Competing interests

The authors declare that they have no competing interests.

## Author’s contributions

JK and BL were responsible for development of the web interface and web-based pipelines. HC launched the GERONTOME project and supervised it. HC provided useful information about the needs of age-related biology research. JK and BL wrote the draft of manuscript. All authors read and approved the final manuscript.
